# Editorial

**Published:** 1901-03-15

**Authors:** 


### EDITORIAL.

February 9th the Detroit Dental Society held its regular monthly meeting at the Detroit College of Medicine, and the afternoon was devoted to an operative clinic by Dr. C. N. Johnson, of Chicago. Dr. Johnson filled with gold a large proximal and occlusal cavity in an upper molar, and not only demonstrated his manipulative methods, but carried out his idea of restoration and protection of the tooth from recurrent decay. The entire operation was a splendid illustration of what may be accomplished when high grade manipulative ability is directed by an intelligent conception of systematic cavity preparation, in facilitating the adaptation of material to meet the sanitary and functional obligation involved. At a banquet the same evening, given in honor of Dr. Johnson, he made an address which revealed the spirit which dominates and has given him the admiration of all who come in contact with him, and has made his work as an instructor of students so successful. He is an earnest and hardworking experimenter as well as an enthusiastic devotee of his art, and combines with this a manly and honorable attitude in judgment toward those who advocate or hold differing principles of practice. His eloquence and enthusiasm as a speaker, while valuable for instruction, must have kindled in the minds of all those present a higher respect and love for their calling, and inspired the younger men, whom he specially addressed, with a profound determination to make the most of every opportunity offering to equip themselves for rendering a high grade of service to their patients.

The paper printed elsewhere in this issue on "Extension for Prevention," by Dr. C. M. Wright, attacks the prevailing practice of sacrificing large quantities of the proximal surfaces of the teeth in the preparation of cavities for the admission of gold, on the ground that the claims made for this method of operating are that fillings can be made more *permanent*. The author criticises the use of this term because it suggests the possibility of individual superiority or bragadocio, and is not calculated to foster scientific researches which make for the discovery of remedial means for overcoming or preventing dental caries, a disease. Consequently it belittles the profession to the sphere of the mechanic. We are not inclined to concede the point that such mechanics as are employed in the production of an artistic gold filling (for illustration) are likely to degrade one's attitude of mind below the highest ideals which must be called into use in the production of a first-class gold filling in a badly decayed tooth. Such work calls for the highest attainments mechanically; not only because of the nature of the material used, but because of the high estimate which intelligent people now place upon their teeth for esthetic as well as beneficial values. Neither does it seem clear to us that the spirit which calls for the most enduring operation is calculated to degrade one's ideals to the level of simple mechanics. The building of a splendid gold filling alone may not call out sufficient inspiration, but to harmonize it with its surroundings, and looking upon it as our best endeavor to restore the Creator's handiwork, makes one more than a mere copyist. He becomes a co-laborer with the Infinite One, and that thought ought to contain sufficient inspiration to lift one far from the possibilities of demeaning himself or his calling, to the highest realms of benevolence. It would be a sad blow to our glory as a profession, to adopt the "*new departure*" methods of temporizing in any of the surgical or technical departments.

---

THE *Ohio Journal* quotes from the *Journal* of the British Dental Association a description of how one Mr. Rippon, solders gold crowns on an "*Asbestos Wig.*" There is nothing particularly novel in the method of soldering the crown, but we are curious to know just what sort of appliance an asbestos wig may be. If any one can enlighten our gloom we shall be relieved and give the description as good a send off as possible in our editorial page. We recall that several years ago our facetious friend, Dr. Wright, introduced the *Wire Bustle* as a swaging block. It has never, so far as we are aware, been copied into our technical nomenclature. But now, if we are to admit the asbestos wig, why not the wire bustle swaging block?

---

THE Ohio, Indiana and Michigan triennial gathering of all the dentists, with their wives or sweethearts, will take place June 4, 5 and 6, 1901, at Indianapolis. This is the third in the series of these gatherings, and it promises to be the most largely attended. At least Indiana is making plans to entertain 800 guests. The program is already well under way and will consist of three papers by the best men available in this and other countries, besides six other papers contributed, two from each of the three States. This will insure a choice selection of papers, and, with so large an attendance, a fine discussion. The main feature, however, of the meeting will be the clinics. It is intended to have the largest number of valuable and entertaining clinics ever seen at any meeting. And besides this there will be social functions galore. We are instructed to make this statement by the committee. We are aware that it smacks a little of exaggeration, but we remember the kind of a time this same committee has provided on the two previous occasions, and we therefore have confidence in its statements. We shall have more to say about it in our next. We advise all our readers to begin now to plan for this meeting.

THE following officers were elected at the recent meeting of the Ohio State Dental Society, at Columbus: President, Dr. H. F. Harvey, of Cleveland; first vice-president, Dr. Otto Arnold, of Columbus; second vice-president, Dr. J. B. Bauman, of Columbus; secretary, Dr. S. D. Ruggles, of Portsmouth; treasurer, Dr. C. I. Keely, of Hamilton.

THE next regular meeting of the Southwestern Michigan Dental Association will be held at Battle Creek, Tuesday and Wednesday, April 9 and 10, 1901. A cordial invitation is extended to all to be present.

C. W. JOHNSON, *Sec'y*.

THE following are the subjects on which candidates for positions as Contract Dental Surgeons in the Army, are examined:

Anatomy, Physiology, Histology, Physics, Chemistry, Metallurgy, Dental Anatomy and Physiology,

Dental Materia Medica and Therapeutics, Dental Pathology and Bacteriology, Orthodontia, Oral Surgery, Operative Dentistry (theoretical), Prosthetic Dentistry (theoretical), Practical Operative Dentistry and Practical Prosthetic Dentistry.

An average of 75 percent will be required in each subject for theoretical branches and 85 percent for practical subjects.

This standard and the personality of the examining board insures good service to the soldiers and will fairly test the necessity for such service.

